# Interaction between temperature and male pheromone in sexual isolation in *Drosophila melanogaster*

**DOI:** 10.1111/jeb.12206

**Published:** 2013-08-14

**Authors:** G Bontonou, B Denis, C Wicker-Thomas

**Affiliations:** CNRS UPR 9034, Université de Paris SudGif sur Yvette, France

**Keywords:** adaptation, desiccation resistance, mating, selection, temperature

## Abstract

In *Drosophila*, female hydrocarbons are known to be involved in premating isolation between different species and pheromonal races. The role of male-specific hydrocarbon polymorphism is not as well documented. The dominant cuticular hydrocarbon (CHC) in male *D. melanogaster* is usually 7-tricosene (7-T), with the exception of African populations, in which 7-pentacosene (7-P) is dominant. Here, we took advantage of a population from the Comoro Islands (Com), in which males fell on a continuum of low to high levels of 7-T, to perform temperature selection and selection on CHCs’ profiles. We conducted several experiments on the selected Com males to study the plasticity of their CHCs in response to temperature shift, their role in resistance to desiccation and in sexual selection. We then compared the results obtained for selected lines to those from three common laboratory strains with different and homogenous hydrocarbon profiles: CS, Cot and Tai. Temperature selection modified the CHC profiles of the Com males in few generations of selection. We showed that the 7-P/7-T ratio depends on temperature with generally more 7-P at higher temperatures and observed a relationship between chain length and resistance to desiccation in both temperature- and phenotypically selected Com lines. There was partial sexual isolation between the flies with clear-cut phenotypes within the phenotypically selected lines and the laboratory strains. These results indicate that the dominant male pheromones are under environmental selection and may have played a role in reproductive isolation.

## Introduction

The evolution of reproductive isolation between divergent conspecific populations is a prerequisite for speciation in animals. Behavioural isolation is one of the most important isolating mechanisms that can lead to speciation, especially sympatric speciation (Coyne & Orr, 2004; Bolnick & Fitzpatrick, 2007). Many *Drosophila* studies have determined the underlying genetics of traits linked to behaviour, such as acoustic or visual signals or contact pheromones (Gleason *et al*., 2005, 2009; Clyne & Miesenböck, 2008; Veltsos *et al*., 2012).

All *Drosophila* species have abundant long-chain hydrocarbons on the surface of their cuticle that could be subject to sexual and natural selection; these cuticular hydrocarbons (CHCs) are involved in chemical communication. Some CHCs function as sex pheromones at short distance or by contact during courtship (Jallon, 1984). CHCs are also subject to natural selection; longer-chain CHCs have a higher melting temperature and are more effective at preventing water loss (Gibbs *et al*., 1997) than shorter-chain CHCs. There is variation in CHC profiles both within and among many *Drosophila* species, but the selective forces that led to this variation are not yet well understood.

The role of female CHCs in sexual isolation between different species and pheromonal races is well established (Wu *et al*., 1995; Coyne, 1996a,b; Coyne & Charlesworth, 1997). There is a geographical CHC polymorphism in *D. melanogaster* females, which affects the composition of the dienes (Ferveur *et al*., 1996). Most female *D. melanogaster* (cosmopolitan populations) produce high levels of 7,11-heptacosadiene (7,11-HD) – a 27C hydrocarbon with two double bonds on carbons 7 and 11 – which acts as the main female pheromone. Females from western Africa, Zimbabwe and the Caribbean produce high levels of a double bond position isomer, 5,9-heptacosadiene (5,9-HD) (Wu *et al*., 1995; Dallerac *et al*., 2000; Takahashi *et al*., 2001).

Several studies have suggested that this female CHC polymorphism led to premating isolation between Zimbabwe and cosmopolitan populations (Begun & Aquadro, 1993; Wu *et al*., 1995; Hollocher *et al*., 1997; Coyne & Elwyn, 2006). One study has suggested that this polymorphism has been driven by temperature; the loss of a functional desaturase in cosmopolitan strains might be involved in resistance to colder temperatures (Greenberg *et al*., 2003). However, this result has not yet been replicated (Coyne & Elwyn, 2006).

The role of male CHCs in sexual isolation has not been as well studied. A geographical CHC polymorphism exists in *D. melanogaster* males. 7-tricosene (7-T) – a 23C hydrocarbon with one double bond in position 7 – is usually the primary male cuticular hydrocarbon. However, males of most African strains produce high levels of 7-pentacosene (7-P) – a 25C hydrocarbon with one double bond in position 7 (Jallon, 1984). 7-T stimulates cosmopolitan females and inhibits male courtship behaviour, whereas 7-P elicits both male–female and male–male courtship in flies from cosmopolitan populations (Jallon, 1984; Antony *et al*., 1985). A recent study has shown that Zimbabwe females were able to discriminate males by the proportion of 7-T in their CHCs, and this has contributed to incipient speciation between Zimbabwe and cosmopolitan strains (Grillet *et al*., 2012). However, there have been no studies on the impact of 7-P on mate choice or speciation. A large-scale study involving 85 *D. melanogaster* populations found a significant correlation between male 7-T/7-P ratio and latitude, mean temperature and vapour pressure (Rouault *et al*., 2001). Moreover, an experimental shift from 18 °C to 29 °C led to increased levels of 7-P and decreased levels of 7-T in three *D. melanogaster* strains (Rouault *et al*., 2004). In the same study, desiccation resistance at 32 °C was higher in Tai, a western African strain, than in Grand-Lieu, a cosmopolitan strain. These data suggest that 7-P and 7-T are involved in heat and desiccation resistance. However, laboratory experiments have not yet clearly demonstrated a link between the evolution of male CHCs and female mate preference in *Drosophila*, which could drive reproductive isolation and ultimately lead to speciation (Rundle, 2003; Rundle *et al*., 2005; Kwan & Rundle, 2010).

Here, we use experimental evolution and artificial selection to investigate the impact of natural and sexual selection on the evolution of male CHCs and, more precisely, the impact of the prevalence of 7-T and 7-P in male CHCs. We took advantage of a unique strain from the Comoro Islands (Com), in which males have heterogeneous hydrocarbon profiles, to perform temperature selection and selection on CHCs’ profiles. As few studies have been conducted on male CHCs, we also decided to use three common laboratory strains with different and homogenous hydrocarbon profiles: CS, Cot and Tai. This allowed us to compare the data obtained for a natural population subjected to manipulations to those from highly inbred strains adapted to laboratory conditions. We studied the role of CHCs in resistance to desiccation and in sexual selection with all strains and lines and also investigated the plasticity of CHCs in response to temperature.

Our results suggest that male CHCs may play a role in sexual selection, which occurs quickly under strong selection on 7-T and 7-P, and that temperature pressure can modify CHC profiles in a few generations, indicating that the response of CHCs to temperature could be a source of the evolution of hydrocarbons in nature.

## Materials and methods

### *Drosophila* strains

We maintained *Drosophila melanogaster* stocks on standard yeast/cornmeal/agar medium in a 12-h-light, 12-h-dark cycle at 25 °C. We used three laboratory strains: CS (Canton-S), a cosmopolitan strain in which males synthesize high levels of 7-T and females high levels of 7,11-HD; Cot (from Cotonou, Benin), in which males synthesize high levels of 7-P and females high levels of 7,11-HD; and Tai (from Tai, Ivory Coast) in which males synthesize high levels of 7-P and females high levels of 5,9-HD. CS and Tai strains have been maintained in the laboratory since the early 1980s and Cot since 2004.

We conducted temperature and pheromone selections on a fourth strain of *D. melanogaster* – Com (from the Comoro Islands).

### *Drosophila* selection lines

**Figure 1:**
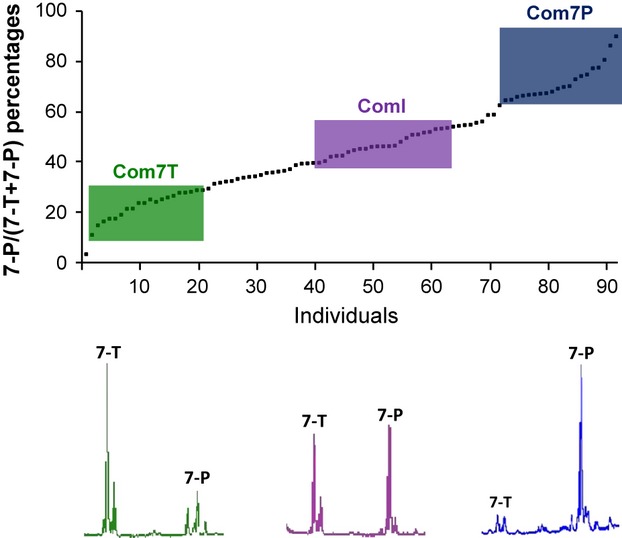
Selection for hydrocarbons in the Com strain. 7-P/(7-T + 7-P) proportions of Com males before selection (F0). Data have been arbitrarily sorted by increasing values. The coloured rectangles represent the 20 males used for selection in the F1 generation (Com7T, ComI and Com7P). Typical chromatograms of cuticular profiles are presented below the curve.

**Figure 2:**
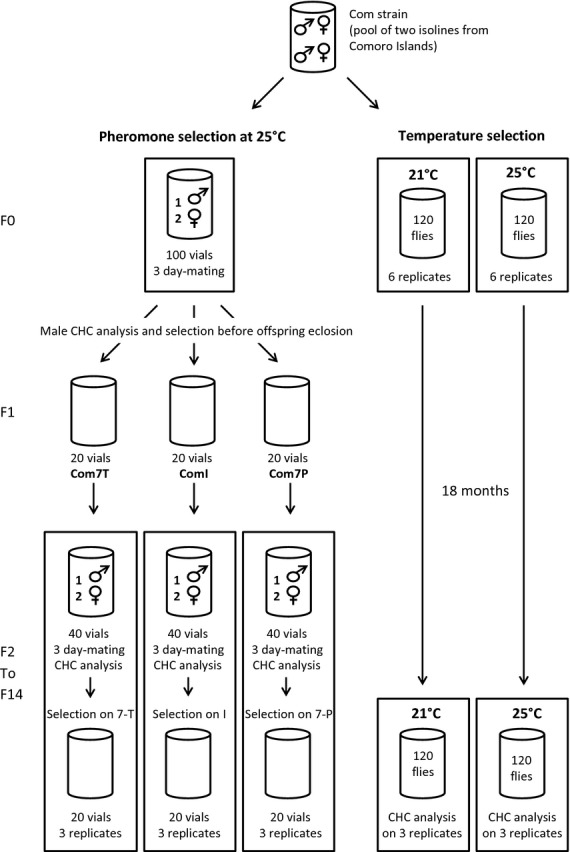
Experimental design for pheromone selection and temperature selection. The initial Com strain was divided into three groups: two groups for the temperature selection and one group for pheromone selection. For the temperature selection, three replicates of two vials were maintained at 25 °C or 21 °C during 18 months. The CHC profiles of male offspring were recorded at the end of the selection process. Pheromone selection was made at 25 °C. Flies were divided into 100 vials containing one male and two females. The CHCs of males were analysed, and the offspring was divided into three groups of 20 vials according to their parental CHCs’ profiles. Virgin collection and round-robin design were carried out as described in text. One replicate was made for the F1 and three replicates from F2 to F14.

Two isofemale lines were collected from Grand Comoro in the Comoro Islands (ML Cariou and M. Schiffer, January 2010). Both isolines presented the same CHC phenotypes: females produced high levels of 7,11-HD, and males fell on a continuum of low to high levels of 7-T (Fig. 1). We mixed both isofemale lines into one strain called Com and divided this strain into three groups for temperature selection and pheromone selection (Fig. 2).

For the temperature selection, we maintained six replicates of 120 flies at either 25 °C or 21 °C. Within each replicate, 60 male and 60 female offspring were used to produce the subsequent generations over 18 months. We recorded the CHC profiles of male offspring at the end of the experiment.

To initiate pheromone selection, we collected 100 virgin males and 200 virgin females from the Com line, separated them by sex and kept them in groups of ten flies on standard medium for 2 days at 25 °C. We then divided the flies into 100 vials, each containing one male and two females. We assigned each tube a number to distinguish the progeny of each male. Flies were allowed to mate and to lay eggs in these vials for 3 days before they were removed, and the vials were incubated at 25 °C during offspring development. We analysed the CHC composition of the 94 males that produced offspring and divided these offspring into three groups: a group of 20 vials from the 20 males with the highest proportion of 7-P (0.70–0.90) called Com7P; a group of 20 vials from the 20 males with the lowest proportion of 7-P (0.10–0.35) called Com7T; and a group of 20 vials from the 20 males with intermediate proportions of 7-P (0.46–0.65) called ComI.

At offspring emergence, we collected virgin males and females from each vial and maintained them on food in groups of ten flies for 2 days at 25 °C. We crossed the flies in a round-robin design with one male and two females within the selection lines. We used a total of 40 vials per replicate to establish the next generation; the males were analysed after mating and the offspring of the 20 males with highest levels of 7-T within the Com7T, the 20 males with highest levels of 7-P within the Com7P and the males with intermediate levels of 7-T and 7-P within the ComI were kept to initiate the next generation. There was one replicate of the F1 generation and three replicates of the F2 to F14 generations. The males with the highest proportion of 7-T were often sterile, and one replicate of Com7T line died at the 8th generation. Selection on CHCs continued to the F14 generation; the F15 and F16 generations were used to conduct the experiments.

### Shift of temperature at emergence

We performed short-term manipulation of temperature to study its impact on male CHCs. The laboratory strains, the Com phenotypically selected lines and the Com25 were bred at 25 °C from egg to emergence. At emergence, males were collected, kept in groups of ten on standard medium and maintained at 25 °C or shifted to 21 °C. The Com21 males were bred at 21 °C from egg to emergence and, at emergence, males were collected and kept in groups of ten on standard medium and maintained at 21 °C or shifted at 25 °C.

Development is temperature dependent, and hydrocarbon maturation takes about 6 days at 21 °C and about 4 days at 25 °C. Therefore, we extracted CHCs on day 7 from individuals reared at 21 °C and on day 5 from individuals reared at 25 °C.

Cuticular hydrocarbons proportions were arcsine square root transformed before normalization. We performed one-way analyses of variance (anovas) separately for each CHC to determine how they are affected by temperature. All statistical analyses were performed using R version 2.13.1 (R Development Core Team, 2011).

### Cuticular hydrocarbon extraction and analyses

We used gas chromatography to analyse the CHC profiles. Each fly was submerged for 5 min in 100 μL of heptane containing 500 ng of hexacosane (C26) as an internal standard. The fly was then removed from the vial, and the vial was sealed with a PTFE (polytetrafluoroethylene) cap to avoid heptane evaporation. All sample extracts were stored at 4 °C prior to gas chromatography.

Five microlitres of each hydrocarbon sample was injected into a Perichrom Pr200 gas chromatograph with hydrogen as the carrier gas, using a split injector (split ratio 40: 1). The column was a 25-m-long BP-1 that had an internal diameter of 220 μm and 0.1 μm film thickness (SGE). The oven temperature started at 180 °C and then ramped at 3 °C min^−1^ to 300 °C, for a total run of 40 min. The flame ionization detector was set at 260 °C.

**Figure 3:**
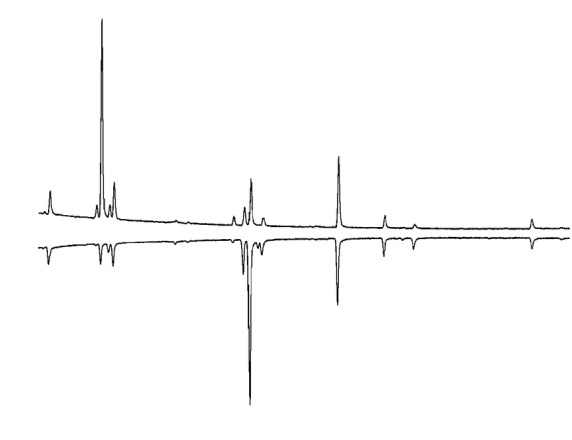
Typical GC profiles of CS (up) and Tai (down) males. The *x*-axis shows the retention time (in minutes) and the *y*-axis the abundance of each peak, in arbitrary units. The first peak is *cis*-vaccenyl acetate, a nonhydrocarbon compound. Peak 12 is the internal standard. The numbers correspond to the compounds listed in Table 1.

We used Winilab III software (version 04.06, Perichrom, Toulouse, France) to carry out the peak integration of hydrocarbon data. We quantified 15 CHCs for each male, with a chain length ranging between 23 and 29 carbons (Table 1). The differences in CHC profiles of cosmopolitan and western African males are illustrated in Fig. 3. We calculated peak areas as a proportion of total CHC content and the total quantities of CHCs by summing the area under each peak and then normalizing this value by dividing by the abundance of the internal standard, C26. We calculated the relative abundance of each CHC by dividing the area under each peak by the total area under all peaks from that individual. This corrected any variation in the extraction of the samples (Blows & Allan, 1998). Data are presented as means ± SEM (*n* = 10 for all tests).

**Table 1:** Identification of the 15 cuticular hydrocarbons in male *Drosophila melanogaster*. The peak numbers correspond to those presented on the chromatogram in Fig. 3. RT is the retention time measured in minutes

Peak	RT	Abbreviation	Chemical formula	Compound name
2	12.32	Me-22	2-Me-C22	2-methyldocosane
3	12.65	9-T	(Z)-9-C23:1	9-tricosene
4	12.81	7-T	(Z)-7-C23:1	7-tricosene
5	13.05	5-T	(Z)-5-C23:1	5-tricosene
6	13.19	C23	C23	tricosane
7	16.86	Me-24	2-Me-C24	2-methyltetracosane
8	17.19	9-P	(Z)-9-C25:1	9-pentacosene
9	17.38	7-P	(Z)-7-C25:1	7-pentacosene
10	17.64	5-P	(Z)-5-C25:1	5-pentacosene
11	17.76	C25	C25	pentacosane
12	20.08	C26	C26	Hexacosane (internal standard)
13	21.5	Me-26	2-Me-C26	2-methylhexacosane
14	22.41	C27	C27	heptacosane
15	26.04	Me-28	2-Me-C28	2-methyloctacosane
16	26.94	C29	C29	Nonacosane

We calculated log-contrasts to compare CHC profiles of males from the selected Com lines at 25 °C. This transformation removed the unit-sum constraint associated with proportional data (Blows & Allan, 1998) and reduced the number of traits by one. We used 2-methyldocosane (Me-22) – a CHC whose proportion does not significantly vary among Com lines at 25 °C – as the denominator (logcontrast(CHC_n_) = log_10_(proportion[CHC_n_]/proportion[Me-22]), resulting in 13 log-contrast variables. We analysed each log-contrast CHC separately using one-way anovas and then used Tukey’s *post hoc* test (including a correction for multiple comparisons) to identify significant differences among groups.

### Desiccation resistance

We performed desiccation experiments within two groups: a first group with the three laboratory strains – CS, Tai and Cot and a second group with selected Com lines – Com7T, ComI and Com7P, and Com21 and Com25. We isolated males at emergence and kept them in groups of 20 in fresh food vials at 25 °C for 5 days. After brief CO_2_ anaesthesia, we transferred 100 males from each experimental strain to empty plastic tubes sealed with a cotton plug that permitted the passage of dry air. We placed these plastic tubes in a hermetically sealed box containing silica gel to maintain low humidity and placed the entire box in an incubator at 25 °C. We recorded mortality every 30 min. We used R (package survival; Therneau & Lumley, 2012) to analyse survival by calculating the median survival time (MST) and its bounds with the survfit function and to test for differences between groups with the surdiff function.

### Mating experiments

We performed mate-choice experiments within the following three groups: a first group with the three laboratory strains – CS, Tai and Cot; a second group with the phenotypically selected Com flies – Com7T, ComI and ComP; and a third group with the Com flies that had been submitted to temperature manipulations, Com21 and Com25.

At emergence, flies were lightly anaesthetized with CO_2_, separated by sex and kept in groups of 10 flies on standard medium for 5 days at 25 °C. We performed courtship assays at approximately the same time each morning at 25 °C. The glass observation chamber used for single-pair tests was a watchglass of 28 mm diameter and 5 mm internal height, placed on a glass plate.

For the *no-choice tests –* which were designed to verify that there was no bias due to different mating interactions when males were mated with females of their own strain – we used the three laboratory strains (CS, Tai and Cot). Males and females from the three strains were paired in the nine possible combinations (*n* = 30 for all tests). We introduced a female into the observation chamber and left her for 1 min before introducing a male. We recorded courtship latency and copulation latency (time between when the male was introduced into the female-containing observation chamber and when courtship or copulation occurred). We used Kruskal–Wallis tests followed by multiple pairwise comparison (pgirmess package; Giraudoux, 2012, kruskalmc function) to compare courtship and copulation latencies between mating types.

For *female-choice tests*, we transferred a single female onto a glass observation chamber along with two males from different strains or lines. The trial ended once the female had copulated with one of the males.

For *multiple-choice tests*, two males and two females (one male and one female of each strain) were introduced into a fresh food vial. The trial ended when the first copulation occurred. We did the multiple-choice tests because they are more sensitive at detecting assortative mating than are no-choice tests, and they allow both male and female choice to contribute to assortative mating (Kwan & Rundle, 2010).

We carried out all assays for female- and multiple-choice tests until 50 copulations had occurred or for 1 h, whichever came first. For statistical purposes, we also recorded the number of flies that did not mate. We cut a small portion of one wing (alternately right or left) of all the flies to allow us to differentiate flies of different strains in mate-choice tests.

For each mate-choice design combination, we used JMating software to calculate the index of sexual isolation (*I*_PSI_; Rolan-Alvarez & Caballero, 2000). *I*_PSI_ is an index that describes overall sexual isolation in experiments. *I*_PSI_ varies from −1 to 1, where −1 represents complete disassortative mating, 0 represents random mating and 1 represents complete assortative mating (complete sexual isolation). We determined statistical significance of *I*_PSI_ by bootstrapping 10 000 times in JMating.

## Results

### Male CHC evolution after pheromone or temperature selection

In January 2010, the two isofemale Com lines exhibited similar hydrocarbon profiles for both males and females. Male pheromonal profiles were heterogenous: 30% of males displayed high amounts of 7-P (proportion of 7-P between 0.70 and 0.90), 30% of males high amounts of 7-T (proportion of 7-P between 0.10 and 0.35) and 40% an intermediate hydrocarbon profile (proportion of 7-P between 0.46 and 0.65) (Fig. 1).

**Figure 4:**
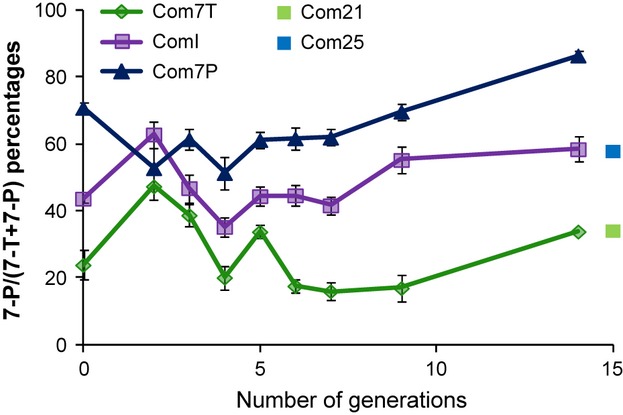
Selection for hydrocarbons in the Com strain. The proportion of 7-P in Com males over 14 generations of selection for Com7T, ComI and Com7P lines at 25 °C. Each value represents mean ± SEM (F0: *n* = 20; F2 to F14: *n* = 2 or 3). Three Com7P lines, three Com7T lines and three ComI lines were initiated during the second selection generation. Data obtained for the three lines are pooled in this figure. Part of Com population has been maintained without selection at 21 °C (noted Com21) or 25 °C (noted Com25) for 18 months and presents 7-T or I to 7-P phenotype, respectively (represented by a green or blue dot).

Throughout the experimental selection process, the replicates showed similar phenotypes within each selection group (Fig. S1). After the 14th generation, the different Com lines globally showed different pheromonal profiles: 7-P, 7-T or I (Fig. 4). However, although 100% of Com7P males synthesized large amounts of 7-P, only 84% of Com7T males synthesized high amounts of 7-T (16% synthesized equal levels of 7-P and 7-T) and 76% of ComI males produced equal levels of 7-P and 7-T (11% produced more 7-T than 7-P and 13% more 7-P than 7-T). The quantitative study of all CHCs indicates that Com7T and Com7P differed essentially in short-chain monoenes (9-T, 7-T, 5-T, 7-P, 5-P, Table 2).

**Table 2:** Analysis of differences between the CHC profiles of males from the Comoro selected lines (Com7T, Com7P, Com21 and Com25) at 25 °C. CHC identities are given in the first column. Me-22 was used as the common divisor in calculating log-contrasts. Statistical analysis was performed using a one-way anova followed by Tukey’s multiple comparison *post hoc* test. *P* values in bold indicate significant CHC variations with strains after multiple test correction. The last four columns give the mean percentages (±SEM) of cuticular hydrocarbons produced by 5-day-old males at 25 °C

CHC	*F*	*P*_Com7T-Com7P_	*P*_Com21-Com25_	*P*_Com7T-Com21_	*P*_Com7P-Com25_	Com7T	Com7P	Com21	Com25
9-T	13.84	**<0.0001**	0.39	**<0.01**	0.17	3.09 ± 0.16	0.5 ± 0.07	2.88 ± 0.21	0.73 ± 0.07
7-T	24.30	**<0.0001**	0.19	**0.03**	**<0.0001**	50.42 ± 0.72	5.82 ± 0.5	37.91 ± 2.07	21.63 ± 0.99
5-T	7.14	**<0.001**	**0.05**	0.17	0.07	4.04 ± 0.11	1.73 ± 0.2	2.18 ± 0.24	1.93 ± 0.19
C23	2.22	0.18	0.09	0.49	0.09	9.48 ± 0.46	7.40 ± 0.70	12.46 ± 0.38	9.83 ± 0.27
Me-24	6.62	**<0.01**	0.17	0.45	**<0.01**	2.29 ± 0.11	0.99 ± 0.12	2.52 ± 0.15	1.76 ± 0.14
9-P	5.44	0.23	**0.02**	0.53	0.87	5.56 ± 0.42	9.12 ± 0.46	6.02 ± 0.37	7.37 ± 0.15
7-P	13.57	**<0.001**	**0.02**	**0.05**	0.29	11.27 ± 0.61	46.21 ± 2.22	20.95 ± 1.75	31.25 ± 1.14
5-P	20.86	**<0.0001**	**0.02**	0.09	**<0.0001**	0.15 ± 0.02	2.36 ± 0.13	0.29 ± 0.03	0.27 ± 0.02
C25	6.49	0.11	**0.02**	0.13	0.41	2.95 ± 0.18	5.39 ± 0.39	4.46 ± 0.25	5.38 ± 0.12
Me-26	5.79	0.45	0.11	0.73	0.26	4.52 ± 0.19	6.40 ± 0.51	4.78 ± 0.19	6.89 ± 0.22
C27	13.74	**0.03**	**<0.001**	0.91	0.43	1.64 ± 0.13	3.92 ± 0.35	1.92 ± 0.15	4.03 ± 0.11
Me-28	8.85	0.70	**0.05**	0.39	0.16	3.31 ± 0.2	4.42 ± 0.49	2.71 ± 0.09	6.06 ± 0.16
C29	10.22	0.79	**0.01**	0.39	**0.04**	0.47 ± 0.08	0.66 ± 0.10	0.42 ± 0.05	1.38 ± 0.14

Part of the Com strain was also maintained at either 21 °C (Com21) or 25 °C (Com25), without selection on CHC profiles. After 18 months, 64% of Com21 males showed high levels of 7-T (36% produced equal levels of 7-P and 7-T), and the Com25 males showed CHC profiles somewhere in between intermediary and 7-P – the proportions of 7-P ranged from 0.46 to 0.82, suggesting that temperature alone was sufficient to drive changes in CHC composition. Further observations on global CHC profiles showed that males from Com21 and Com25 differed by C25 monoenes (9-T and 7-P) but also by C27 and C29 saturated (linear and branched) CHCs (Table 2).

We then compared the CHC profiles of male flies produced through the two different selection regimes (temperature and pheromone selection) to see whether the resulting flies shared similar CHC profiles. Both selections led to similar CHC profiles: Com7T and Com21 males significantly differed by only three CHCs (9-T, 7-T and 7-P), whereas Com7P and Com25 flies differed by four CHCs (7-T, Me-24, 5-P and C29; Table 2).

### Male CHC plasticity in response to a shift of temperature at emergence

**Figure 5:**
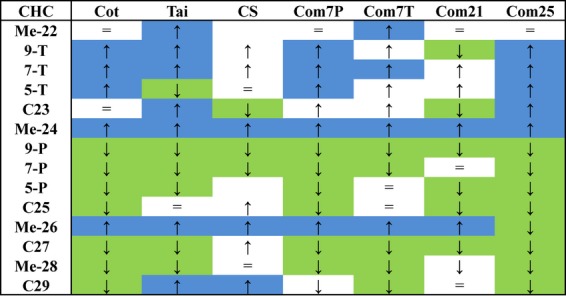
Impact of a temperature shift from emergence on the CHCs of different strains and lines. Arrows indicate an increase or decrease in the relative abundance of individual CHCs. Blue and green colours indicate statistically significant variations.

Whatever the temperature, the proportions of different CHC classes were about the same in all the strains and lines: 75% monoenes, 15% linear and 10% branched. However, the different temperatures led to different changes in specific hydrocarbons, depending on the line (Fig. 5).

Cuticular hydrocarbons of the two laboratory strains with high levels of 7-P (Cot and Tai) responded similarly to temperature: the percentages of most CHCs with 23 carbons, of Me-24 and of Me-26 were higher in those flies maintained 7 days at 21 °C than in those maintained 5 days at 25 °C. The other CHCs decreased at 21 °C (Table S1). 7-T and 7-P, which constitute more than 50% of the total CHCs, were particularly affected; there was a 100% increase in 7-T in both Cot and Tai and a 22% and 14% decrease in 7-P, respectively (Fig. S2). In CS, the laboratory strain with high levels of 7-T, percentages of Me-24 and of Me-26 were multiplied by 200% and 100% at 21 °C, whereas C23 and 7-P decreased by 28% and 58%, respectively.

**Figure 6:**
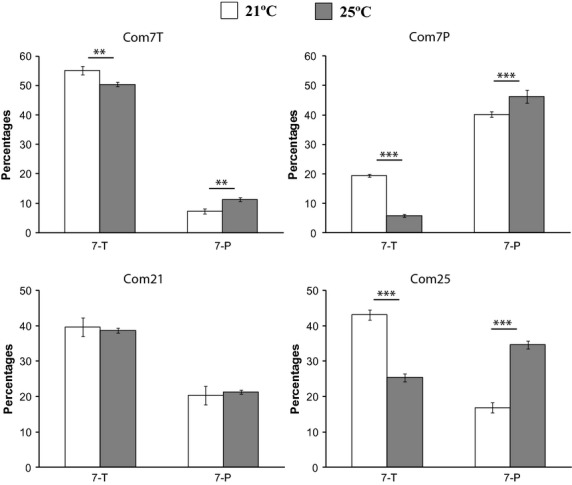
Effect of temperature (5 days at 25 °C or 7 days at 21 °C) on the relative levels of 7-T and 7-P in Com7T, Com7P, Com21 and Com25 lines. Each bar represents mean ± SEM (*n* = 10). *, ** and *** above bars indicate significant differences (one-way anova, *P* = 0.05, 0.01 and 0.001, respectively) between means.

Cuticular hydrocarbon profiles of males from the Com phenotypically selected lines responded to temperature modifications similarly to those of laboratory males. Com7P males showed the same CHC variation as Cot and Tai, with a strong effect on the percentages of 7-T and 7-P (Fig. 6); they had 225% more 7-T and 19% less 7-P at 21 °C than at 25 °C. Com7T males had 9% more 7-T, 49% less 9-P and 35% less 7-P at 21 °C, similar to CS males There was also more variation in linear and branched CHCs: 58% more Me-22, 89% more Me-24, 20% more Me-26, 37% less Me-28 and 49% less C29 (Table S2).

The temperature-selected lines, Com21 and Com25, showed a peculiar response to a shift in temperature. There was no modification of the 7-T and 7-P amounts of Com21 males when they were shifted from 21 °C to 25 °C (*F*_1,39_ = 0.04, *P* = 0.84 and *F*_1,39_ = 0, *P* = 0.99, respectively; Fig. 6, Table S3). The compounds that were more affected were the linear and branched CHCs (-21% C23, +61% Me-26). In contrast, when Com25 males were transferred to 21 °C, all their CHCs were significantly affected. Moreover, their 7-P profile (7-P/(7-T + 7-P) = 0.6) was transformed into an essentially 7-T profile (7-P/(7-T + 7-P) = 0.28).

### Role of CHCs in desiccation resistance

**Figure 7:**
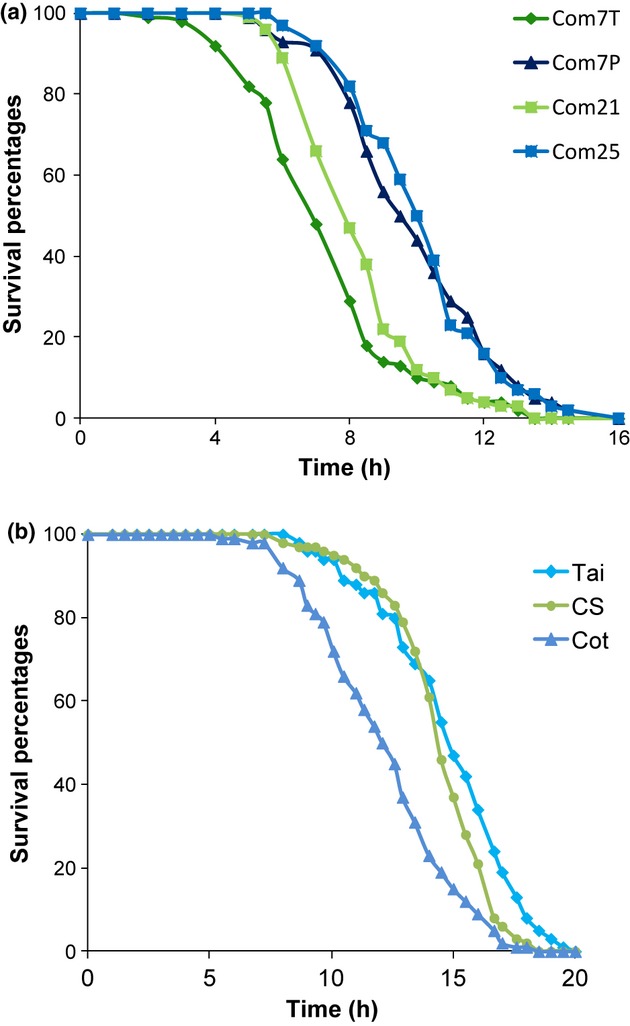
Survival proportions of male flies from different strains, as a function of exposure time to desiccation stress at 25 °C (*n* = 100). (a) Desiccation profiles of male flies from Com7T, Com7P, Com21 and Com25 lines. (b) Desiccation profiles of male flies from Tai, CS and Cot strains.

There was a positive correlation between CHC chain length and desiccation resistance in the temperature- and phenotypically selected Com lines: Com7T and Com21 were less resistant (MST values of 7 h and 8 h, respectively) than Com7P and Com25 (MST values of 9 h and 10 h, respectively), which displayed less 7-T, but more 7-P and more CHCs in 27 and 29C (Fig. 7a; Table 1; Table S4). Com7T was significantly less resistant than Com21 (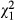 = 6.7, *P* = 0.01), which had less 7-T and more 7-P, with no change in longer CHCs. Com7P and Com25 showed similar resistance to desiccation (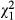 = 0.1, *P* = 0.79), although Com25 had three times more 7-T and a few more linear and branched CHCs.

However, there was no correlation between CHC composition and desiccation resistance in the three laboratory strains (Fig. 7b). Cot males had higher levels of 7-P than CS males and higher levels of CHCs with 27 of 29 carbons than both CS and Tai males, but they were the least resistant to desiccation (MST values of 12 h20, 14 h30 and 15 h for Cot, CS and Tai, respectively).

### Mate-choice experiments

#### Laboratory strains

In no-choice tests, males and females from CS, Cot and Tai were paired in all nine possible combinations. Ninety five to 99% of mating pairs copulated within 40 min. Intrastrain courtship latency varied significantly among the three laboratory strains (Kruskal–Wallis χ² = 13.5, *P* = 0.001), because Tai males began to court later than the other males. Intrastrain copulation latency was similar among the three laboratory strains (Kruskal–Wallis χ² = 4.75, *P* > 0.05) (Figure S3a,b). Courtship and copulation latency varied significantly among the nine crosscombinations of the strains (Kruskal–Wallis χ² = 57, *P* < 0.001 and Kruskal–Wallis χ² = 76, *P* < 0.001). For each combination, male courtship latency was independent of the female pheromone profile (Kruskal–Wallis χ² = 1.65, *P* > 0.05 for crosses with a Tai male, Kruskal–Wallis χ² = 5.44, *P* > 0.05 for crosses with a CS male and Kruskal–Wallis χ² = 4.68, *P* > 0.05 for crosses with a Cot male). Copulation latency varied according to the male CHC profiles. Copulation latency was two to three times higher with a Cot or a Tai male than with a CS male because, although Cot and Tai males persistently courted, they were not accepted by the female. Tai males mated at least twice as quickly with Tai females (8 min) than with CS (24 min) or Cot ones (16 min) (Fig. S3b). In female-choice tests, both Cot and Tai females preferred their own males over CS males. CS females mated more frequently with Tai males, probably due to the CS male behaviour; CS males persistently courted the other 7-P male, giving the Tai male more opportunities to copulate. This could also explain why CS females mated in the same proportions with CS and Cot males. To quantify the degree of sexual isolation, we calculated the sexual isolation index (*I*_PSI_) for each cross. The distribution of mating revealed positive and significant sexual isolation between CS and Tai and between CS and Cot in both female- and multiple-choice experiments. Asymmetric sexual isolation was observed in female-choice experiment with Cot and Tai, due to Tai female preference for Cot males (Table 3; Fig. S4a). This preference disappeared in multiple-choice experiments (Table 4; Fig. S4b). Cot females did not distinguish between Cot and Tai males in either experiment.

**Table 3:** Number of observed pair matings and estimates of sexual isolation across experimental designs. One female is given the choice between two males. In pairings, identity of female is given first. Female choices are compared for each cross. *I*_PSI_ coefficients, their standard deviations and their significance of deviation from the null hypothesis (i.e. random mating) were calculated in JMATING by resampling the observed values 10 000 times

Number of replicates	Number of effective matings				*I*_PSI_ ± 1SD	*P*
100	CS CS	CS Tai	Tai CS	Tai Tai	0.40 ± 0.10	**<0.001**
	15	35	3	47		
100	CS CS	CS Cot	Cot CS	Cot Cot	0.23 ± 0.10	**0.026**
	26	24	15	35		
121	Cot Cot	Cot Tai	Tai Cot	Tai Tai	0.33 ± 0.10	**0.002**
	27	23	41	9		
122	Com7T Com7T	Com7T Com 7P	Com7P Com7T	Com7P Com7P	0.23 ± 0.10	**0.025**
	36	14	25	25		
160	ComI ComI	ComI Com7P	Com7P ComI	Com7P Com7P	−0.06 ± 0.10	0.541
	24	26	27	23		
156	Com7T Com7T	Com7T ComI	ComI Com7T	ComI ComI	−0.02 ± 0.10	0.844
	28	22	21	29		
132	Com 21 Com 21	Com 21 Com 25	Com 25 Com 21	Com 25 Com 25	0.10 ± 0.10	0.329
	30	20	25	25		

Bold values are significance.

**Table 4:** Number of observed pair matings and estimates of sexual isolation across experimental designs. Two males and two females (one male and one female of two strains) were put together. In pairings, species of female is given first. Multiple choices are compared for each cross. *I*_PSI_ coefficients, their standard deviations and their significance of deviation from the null hypothesis (i.e. random mating) were calculated in JMATING by resampling the observed values 10 000 times

Number of replicates	Number of effective matings				*I*_PSI_ ± 1SD	*P*
62	CS CS	CS Tai	Tai CS	Tai Tai	0.34 ± 0.12	**0.009**
	20	12	8	22		
55	CS CS	CS Cot	Cot CS	Cot Cot	0.55 ± 0.12	**<0.001**
	15	7	6	27		
52	Cot Cot	Cot Tai	Tai Cot	Tai Tai	0.16 ± 0.15	0.298
	17	11	10	12		
53	Com7T Com7T	Com7T Com 7P	Com7P Com7T	Com7P Com7P	0.32 ± 0.14	**0.025**
	14	8	9	19		
55	ComI ComI	ComI Com7P	Com7P ComI	Com7P Com7P	0.18 ± 0.14	0.241
	15	14	13	8		
51	Com7T Com7T	Com7T ComI	ComI Com7T	ComI ComI	−0.04 ± 0.15	0.770
	12	12	14	12		
54	Com 21 Com 21	Com 21 Com 25	Com 25 Com 21	Com 25 Com 25	0.11 ± 0.15	0.445
	9	14	9	22		

Bold values are significance.

#### Selected Com7T and Com7P lines

Female-choice and multiple-choice tests revealed sexual isolation between the Com7T and Com7P selected lines after 14 generations (*I*_PSI_ = 0.234, *P* = 0.025 and *I*_PSI_ = 0.320, *P* = 0.025, respectively; Tables 3 and 4; Fig. S3). Females frequently mated with males of their own line, and Com7T females appeared to be more discriminatory than were Com7P females in female-choice experiments. *I*_*PSI*_ values did not significantly differ from 0 when mating tests were performed between ComI and either Com7T or Com7P flies.

#### Com21 and Com25 lines

No premating isolation was observed between Com21 and Com25 lines after 18 months (*I*_PSI_ = 0.103, *P* = 0.329 for female-choice test and *I*_PSI_ = 0.113, *P* = 0.445 for multiple-choice test) even though Com21 females mated more frequently with Com21 males than with Com25 males in female-choice experiments (30 effective matings vs. 20). These results show that different male pheromone profiles are associated with partial sexual isolation between lines or strains. Moreover, temperature pressure was able to modify CHC profiles in a few generations but these changes were not still sufficient to induce sexual isolation.

## Discussion

This study suggests that male CHCs play a role in both mating behaviour and adaptation to an environmental stress. In all strains and lines, we observed variation in CHCs in response to temperature that generally resulted in an increase in long-chain CHCs.

Reproductive isolation between cosmopolitan strains and African strains has been described (Coyne & Elwyn, 2006; Grillet *et al*., 2012), but has been linked to female CHC profiles (Wu *et al*., 1995; Dallerac *et al*., 2000; Takahashi *et al*., 2001). The role of male-specific hydrocarbon polymorphism is not as well documented. Here, we demonstrate sexual isolation between a cosmopolitan strain (CS: 7-T males and 7,11-HD females) and two African strains (Cot and Tai: 7-P males and 7,11-HD or 5,9-HD females), suggesting the importance of female choice in 7-T or 7-P mate recognition. This importance is confirmed by our results on Com selected lines; selection for pheromone phenotypes led to partial sexual isolation between Com7T and Com7P flies in only 14 generations. The role of male pheromone differences in sexual isolation has also been shown in the pheromonal races of the closely related species *D. simulans* (Bontonou *et al*., 2012) and in most distant species; selection on CHCs in *D. serrata* has been shown to result in sexual isolation in only nine generations (Higgie *et al*., 2000). This confirms that pheromones are important factors that can lead to rapid sexual isolation in *Drosophila*.

Male pheromone composition and female preference involve different genes (Lofstedt *et al*., 1989) and evolve independently (Rundle *et al*., 2009), but selection on female choice has been shown to cause evolution of male CHCs in *D. serrata* (Higgie & Blows, 2007). There is no correlation between expression of *desat1* – the only described gene known to be involved in both the emission and the perception of sex pheromones in *Drosophila* (Bousquet *et al*., 2012) – and the mating patterns of *Drosophila* strains (Grillet *et al*., 2012).

CHCs can also be modified by the ecological conditions experienced by flies in nature. *D. mojavensis* populations are highly dependent on different cactus host plants that may have driven CHC differences involved in sexual isolation between populations (Etges *et al*., 2009). A prereproductive isolation has also been described between two *D. melanogaster* populations from Brazzaville (Capy *et al*., 2000). These populations live in different ecological environments, and pheromone and microsatellite studies suggest that one population might have originated from Europe and that reproductive isolation between both populations has been maintained partly because they do not share the same habitat (Haerty *et al*., 2005). Among the many different strains we have analysed for cuticular hydrocarbons shortly after they were collected, Com is the only one that presents a continuum between low and high levels of 7-T. Both lines that formed the Com strain originated from one female and showed identical phenotypes, with a continuum of male phenotypes between high levels of 7-T and high levels of 7-P. Presumably, these phenotypes share the same ecological niches.

We failed to observe sexual isolation between the Com lines selected by temperature. However, although Com 25 females showed no preference, Com21 females preferred Com21 males. Temperature selection may take more generations than occurred in our study to induce clear-cut male CHC phenotypes that would result in sexual isolation. This lack of sexual isolation in the temperature-selected lines could be explained by the male hydrocarbon profiles. Sexual isolation was observed only when males displayed clear-cut phenotypes, and the CHC profile of Com25 males was between ComI and Com7P profiles. Another study also failed to generate sexual isolation between laboratory populations selected for different resistance to desiccation (Kwan & Rundle, 2010).

*Drosophila* CHCs originate from unsaturated fatty acids, which are elongated to form very long unsaturated fatty acids, which are then decarboxylated (Dallerac *et al*., 2000). The elongation of fatty acids to C24 or C26 fatty acids, precursors of tricosene and pentacosene, might be under temperature regulation. The CHC adaptation to temperature parallels an adaptation to desiccation resistance in the Com lines, but no correlation was observed between CHC length and desiccation resistance in laboratory strains. Quantitative genetic studies suggest that there is an association between CHC composition and desiccation survival in *D. melanogaster* (Foley & Telonis-Scott, 2011), and selection for resistance to desiccation is accompanied by changes in HC pattern, generally an increase in chain length (Kwan & Rundle, 2010; Foley & Telonis-Scott, 2011). Other studies have noted the importance of additional parameters such as water loss, water content (Gibbs *et al*., 1997; Bazinet *et al*., 2010), lipids (Clark & Doane, 1983; Van Herrewege & David, 1997) and carbohydrates (Graves *et al*., 1992; Chippindale *et al*., 1998; Gefen *et al*., 2006). CS and Tai laboratory strains have been kept in the laboratory for several decades, and this adaptation to a very stable environment has led to increased weight and resistance to desiccation (the median survival time is more than 14 h), and Cot flies, which have been kept for less than a decade in the laboratory, are still smaller, have smaller amounts of hydrocarbons and are less resistant to desiccation than CS and Tai. The Com flies, which are relatively new, are the less resistant to desiccation (median survival times comprised between 7 h and 10 h). These flies, which belong to the same strain, are more genetically close than the flies from different laboratory strains, and a correlation between CHC length and resistance to desiccation was observed. The adaptation of the strains to the laboratory might have increased their fat content, resulting in increased resistance to desiccation. We have recently shown that the overexpression of a lipocalin gene, *lazarillo*, caused starvation-desiccation resistance, due to fat storage promotion without modification of CHC profiles or quantities (Ruiz *et al*., 2012).

This pheromone heterogeneity in Com flies raises several questions: Did these flies arrive recently or are they a remnant of an ancestral strain that evolved on this island in isolation? The strong correlation between 7-P content and resistance to desiccation in the four Com lines would suggest a common genetic background. Conditions in nature are much more variable than in the laboratory (temperature, food, etc.), and the polymorphism could provide an advantage in changing environmental conditions. It would be very useful to collect flies in Comoro Islands over the next few years to see whether the phenotype changes over time in nature.

## Supporting Information

Figure S1 Selection for hydrocarbons in the different Com lines.

Figure S2 Effect of a temperature shift at emergence (7 days from 25 to 21 °C or 5 days from 25) on the absolute quantities of hydrocarbons and on the relative levels of 7-T and 7-P in Canton-S (CS), Cotonou (Cot) and Tai.

Figure S3 Laboratory strains, CS, Cot and Tai, show sexual isolation.

Figure S4 (a) Female-choice mating experiments. On the *X*-axis, 1–2 indicates that female from strain 1 is given the choice between two males (one male from strain1 and one male from strain 2).

Table S1 Analysis of differences between the CHC profiles of males from Cot, Tai and CS at two temperatures.

Table S2 Analysis of differences between the CHC profiles of males from Com7T and Com7P at two temperatures.

Table S3 Analysis of differences between the CHC profiles of males from Com21 and Com 25 at two temperatures.

Table S4 Resistance against desiccation in Com lines and in laboratory strains.
