# Supplementary figures and images for: Interaction between temperature and male pheromone in sexual isolation in Drosophila melanogaster
Source: J Evol Biol. 2013 Aug 14;26(9):2008–20. doi: 10.1111/jeb.12206 (PMC4217391; doi:10.1111/jeb.12206)

7-P/(7-T+7-P) percentages

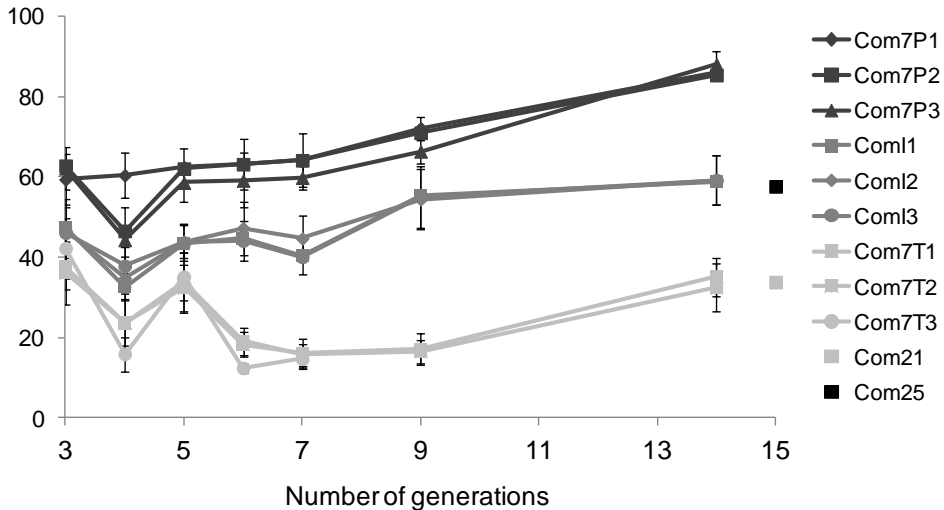

Supplement: Figure S1 — Selection for hydrocarbons in the different Com lines. [file jeb0026-2008-sd1.pdf]

**Cot**

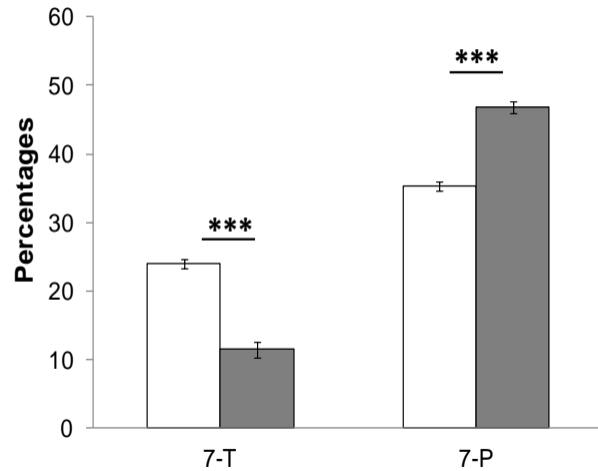

**Tai**

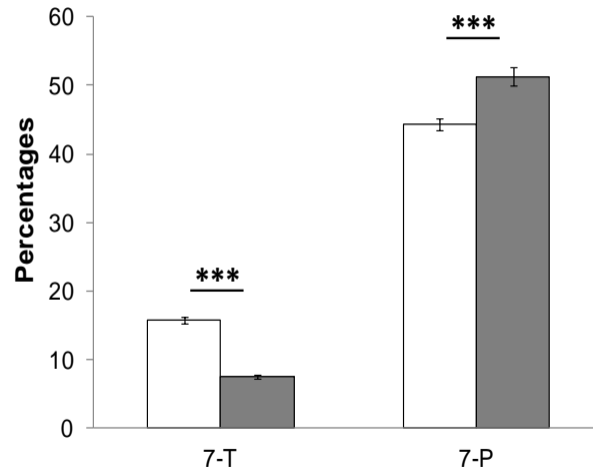

**CS**

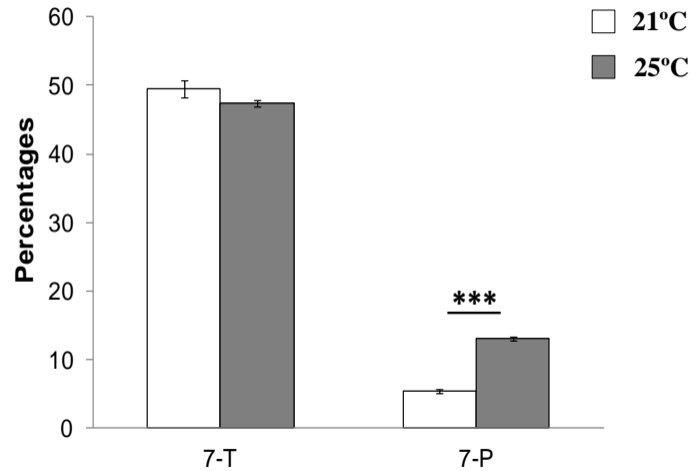

Supplement: Figure S2 — Effect of a temperature shift at emergence (7 days from 25 to 21 °C or 5 days from 25) on the absolute quantities of hydrocarbons and on the relative levels of 7-T and 7-P in Canton-S (CS), Cotonou (Cot) and Tai. [file jeb0026-2008-sd2.pdf]

A

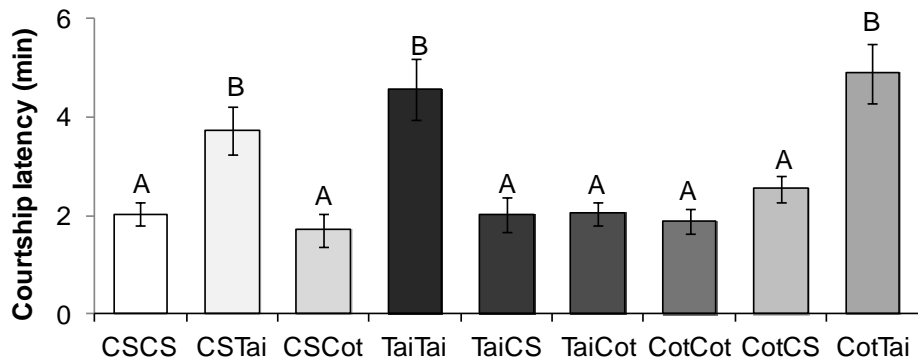

B

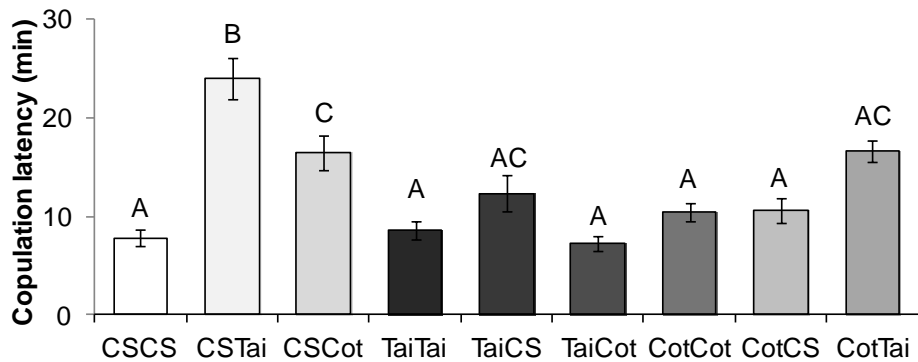

Supplement: Figure S3 — Laboratory strains, CS, Cot and Tai, show sexual isolation. [file jeb0026-2008-sd3.pdf]

A

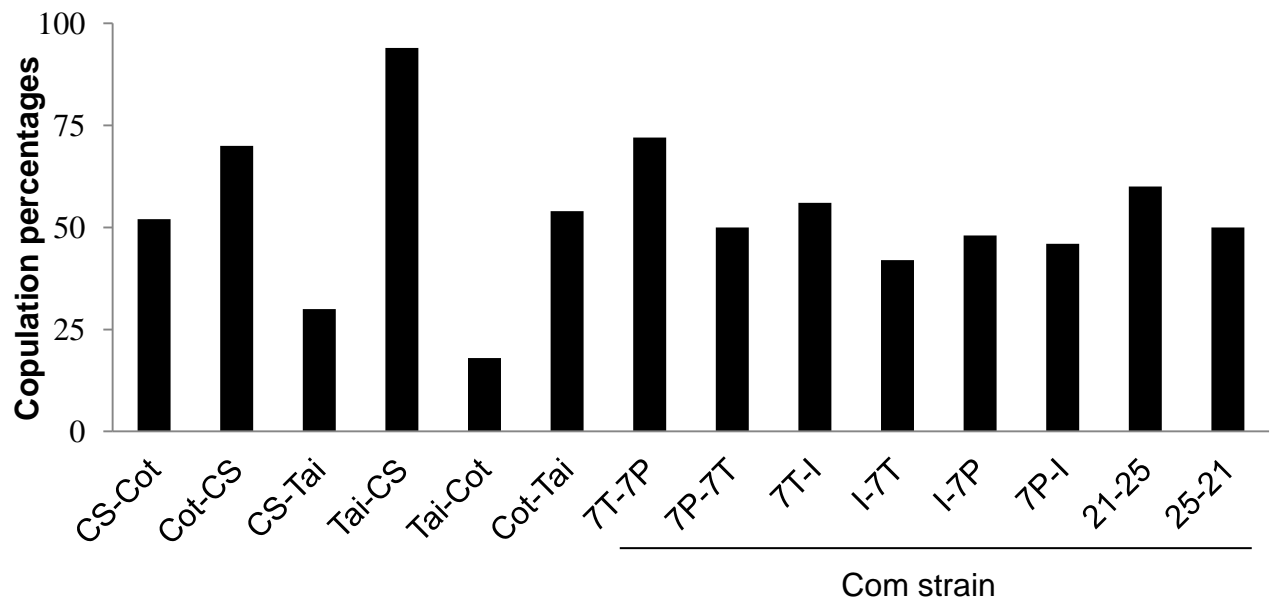

B

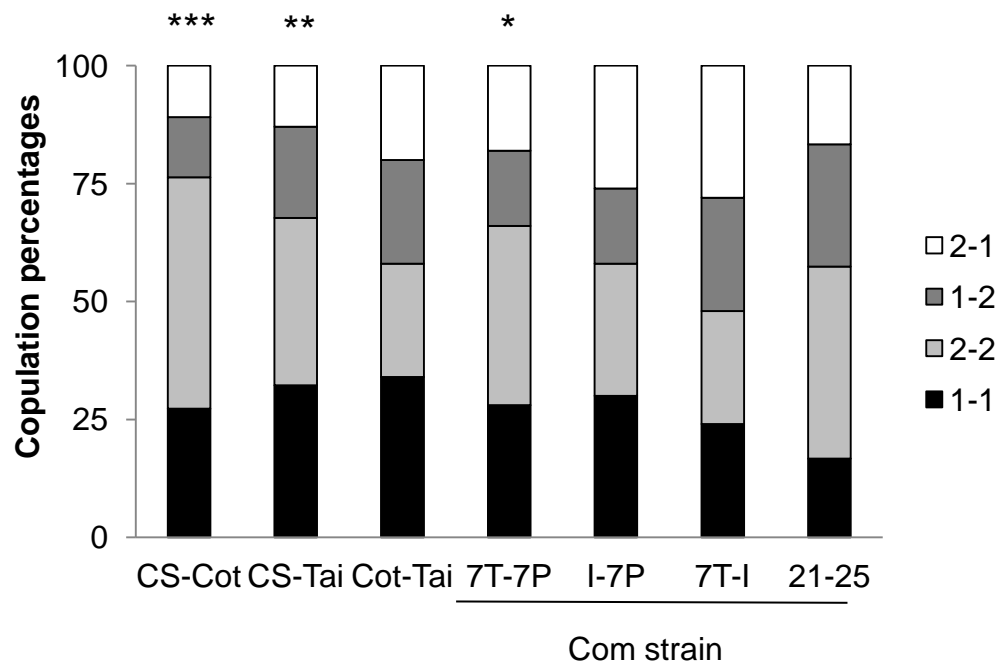

Supplement: Figure S4 — (a) Female-choice mating experiments. On the X-axis, 1–2 indicates that female from strain 1 is given the choice between two males (one male from strain1 and one male from strain 2). [file jeb0026-2008-sd4.pdf]
