# Supplementary material for: Interaction between temperature and male pheromone in sexual isolation in Drosophila melanogaster
Source: J Evol Biol. 2013 Aug 14;26(9):2008–20. doi: 10.1111/jeb.12206 (PMC4217391; doi:10.1111/jeb.12206)
Supplement: Table S2 — Analysis of differences between the CHC profiles of males from Com7T and Com7P at two temperatures. [file jeb0026-2008-sd6.docx]

Table S2. Analysis of differences between the CHC profiles of males from Com7T and Com7P at two temperatures. CHC identities are given in the first column. Statistical analysis was performed using a one-way ANOVA. Values in bold indicate significant CHC variations with temperature. The last two columns give the mean percentage (± SEM) of cuticular hydrocarbons produced by individual 7-day-old males at 21°C or 5-day-old males at 25°C.

|  | **Com7T** | | | | **Com7P** | | | |
| --- | --- | --- | --- | --- | --- | --- | --- | --- |
| **CHC** | ***F*** | ***P*** | **21°C** | **25°C** | ***F*** | ***P*** | **21°C** | **25°C** |
| **Me-22** | 7.29 | 0.02 | 0.27 ± 0.03 | 0.17 ± 0.02 | 2.50 | 0.13 | 0.13 ± 0.02 | 0.11 ± 0.05 |
| **9-T** | 2.37 | 0.14 | 3.69 ± 0.33 | 3.09 ± 0.16 | 109.20 | **<.0001** | 1.21 ± 0.08 | 0.51 ± 0.06 |
| **7-T** | 8.73 | **<.01** | 55.16 ± 1.43 | 50.42 ± 0.71 | 293.77 | **<.0001** | 19.40 ± 0.50 | 5.97 ± 0.43 |
| **5-T** | 2.91 | 0.11 | 4.33 ± 0.14 | 4.03 ± 0.10 | 33.80 | **<.0001** | 3.13 ± 0.10 | 1.76 ± 0.18 |
| **C23** | 0.82 | 0.38 | 9.95 ± 0.30 | 9.48 ± 0.45 | 1.33 | 0.26 | 8.13 ± 0.24 | 7.62 ± 0.63 |
| **Me-24** | 23.09 | **<.0001** | 4.34 ± 0.29 | 2.29 ± 0.11 | 69.82 | **<.0001** | 2.61 ± 0.17 | 1.01 ± 0.10 |
| **9-P** | 29.64 | **<.0001** | 2.85 ± 0.30 | 5.55 ± 0.42 | 78.62 | **<.0001** | 4.89 ± 0.22 | 9.44 ± 0.39 |
| **7-P** | 14.18 | **<.01** | 7.25 ± 0.93 | 11.27 ± 0.61 | 5.59 | 0.03 | 40.18 ± 0.91 | 49.65 ± 1.75 |
| **5-P** | 0.98 | 0.34 | 0.14 ± 0.04 | 0.14 ± 0.02 | 90.96 | **<.0001** | 1.12 ± 0.06 | 0.61 ± 0.11 |
| **C25** | 0.48 | 0.50 | 2.75 ± 0.38 | 2.95 ± 0.18 | 18.65 | **<.001** | 3.64 ± 0.17 | 5.59 ± 0.37 |
| **Me-26** | 4.60 | 0.05 | 5.42 ± 0.38 | 4.51 ± 0.19 | 23.38 | **<.001** | 9.58 ± 0.40 | 6.58 ± 0.43 |
| **C27** | 5.06 | 0.04 | 1.11 ± 0.25 | 1.64 ± 0.12 | 39.17 | **<.0001** | 1.47 ± 0.22 | 4.04 ± 0.31 |
| **Me-28** | 17.41 | **<.001** | 2.07 ± 0.23 | 3.31 ± 0.20 | 4.50 | 0.05 | 3.05 ± 0.43 | 4.56 ± 0.49 |
| **C29** | 6.272 | 0.02 | 0.24 ± 0.08 | 0.47 ± 0.08 | 0.46 | 0.51 | 0.60 ± 0.09 | 0.66 ± 0.10 |
