# Supplementary material for: Interaction between temperature and male pheromone in sexual isolation in Drosophila melanogaster
Source: J Evol Biol. 2013 Aug 14;26(9):2008–20. doi: 10.1111/jeb.12206 (PMC4217391; doi:10.1111/jeb.12206)
Supplement: Table S3 — Analysis of differences between the CHC profiles of males from Com21 and Com 25 at two temperatures. [file jeb0026-2008-sd7.docx]

|  | **Com21** | | | | **Com25** | | | |
| --- | --- | --- | --- | --- | --- | --- | --- | --- |
| **CHC** | ***F*** | ***P*** | **21°C** | **25°C** | ***F*** | ***P*** | **21°C** | **25°C** |
| **Me-22** | 2.01 | 0.16 | 0.25 ± 0.04 | 0.21 ± 0.03 | 1.06 | 0.52 | 0.14 ± 0.02 | 0.10 ± 0.02 |
| **9-T** | 29.78 | **<.0001** | 1.42 ± 0.09 | 2.88 ± 0.21 | 129.29 | **<.0001** | 1.65 ± 0.08 | 0.73 ± 0.07 |
| **7-T** | 0.04 | 0.84 | 39.21 ± 1.31 | 37.91 ± 2.07 | 373.71 | **<.0001** | 44.94 ± 0.83 | 21.63 ± 0.99 |
| **5-T** | 2.55 | 0.11 | 2.55 ± 0.14 | 2.18 ± 0.24 | 13.57 | **<.001** | 2.75 ± 0.16 | 1.93 ± 0.19 |
| **C23** | 26.46 | **<.0001** | 9.89 ± 0.30 | 12.46 ± 0.38 | 32.92 | **<.0001** | 11.90 ± 0.25 | 9.83 ± 0.27 |
| **Me-24** | 133.08 | **<.0001** | 5.47 ± 0.21 | 2.52 ± 0.15 | 164.45 | **<.0001** | 4.20 ± 0.13 | 1.76 ± 0.14 |
| **9-P** | 4.14 | **0.05** | 4.60 ± 0.21 | 6.02 ± 0.37 | 203.32 | **<.0001** | 4.23 ± 0.16 | 7.37 ± 0.15 |
| **7-P** | 0,000 | 0.99 | 20.36 ± 1.33 | 20.95 ± 1.75 | 112.55 | **<.0001** | 17.34 ± 0.71 | 31.25 ± 1.14 |
| **5-P** | 22.14 | **<.0001** | 0.19 ± 0.02 | 0.29 ± 0.03 | 8.46 | **<.01** | 0.22 ± 0.01 | 0.27 ± 0.02 |
| **C25** | 4.17 | **0.05** | 3.75 ± 0.15 | 4.46 ± 0.25 | 137.9 | **<.0001** | 3.73 ± 0.09 | 5.38 ± 0.12 |
| **Me-26** | 67.50 | **<.0001** | 7.71 ± 0.28 | 4.78 ± 0.19 | 18.46 | **<.0001** | 5.86 ± 0.18 | 6.89 ± 0.22 |
| **C27** | 14.63 | **<.001** | 1.18 ± 0.08 | 1.92 ± 0.15 | 500.52 | **<.0001** | 1.17 ± 0.06 | 4.03 ± 0.11 |
| **Me-28** | 3.28 | 0.07 | 2.41 ± 0.17 | 2.71 ± 0.09 | 802.68 | **<.0001** | 1.49 ± 0.09 | 6.06 ± 0.16 |
| **C29** | 0.32 | 0.58 | 0.39 ± 0.04 | 0.42 ± 0.05 | 302.15 | **<.0001** | 0.30 ± 0.02 | 1.38 ± 0.14 |

Table S3. Analysis of differences between the CHC profiles of males from Com21 and Com25 at two temperatures. CHC identities are given in the first column. Statistical analysis was performed using a one-way ANOVA. Values in bold indicate significant CHC variations with temperature. The last two columns give the mean percentage (± SEM) of cuticular hydrocarbons produced by individual 7-day old males at 21°C or 5-day old males at 25°C.
