# Supplementary material for: Interaction between temperature and male pheromone in sexual isolation in Drosophila melanogaster
Source: J Evol Biol. 2013 Aug 14;26(9):2008–20. doi: 10.1111/jeb.12206 (PMC4217391; doi:10.1111/jeb.12206)
Supplement: Table S4 — Resistance against desiccation in Com lines and in laboratory strains. [file jeb0026-2008-sd8.docx]

Table S4. Resistance against desiccation in Com lines and in laboratory strains. Hydrocarbon amounts are expressed in ng/ fly (mean ± SEM); n=10. Resistance against desiccation was performed on 100 male flies at 25°C. MST (median survival time) were computed based on 100 flies and expressed in hours. 95% confidence intervals of the MST are included.

| Line | Type | HC | Lower | MST | Upper |
| --- | --- | --- | --- | --- | --- |
| Com7T | 7-T | 1665± 133 | 7h00 | 7h00 | 8h00 |
| Com7P | 7-P | 1925± 182 | 9h00 | 9h45 | 10h30 |
| Com21 | 7-T | 1314 ± 92 | 8h00 | 8h00 | 8h30 |
| Com25 | I to 7-P | 1761 ± 143 | 10h00 | 10h15 | 10h30 |
| CS | 7-T | 1845 ± 100 | 14h30 | 14h30 | 15h00 |
| Cot | 7-P | 1527 ± 150 | 11h20 | 12h20 | 12h55 |
| Tai | 7-P | 1817 ± 161 | 14h30 | 15h00 | 16h00 |
